# Supplementary material for: Electrochemical Impedance Spectroscopy for the Sensing of the Kinetic Parameters of Engineered Enzymes
Source: Sensors (Basel). 2024 Apr 20;24(8):2643. doi: 10.3390/s24082643 (PMC11054234; doi:10.3390/s24082643)
Supplement: Supplementary file 1 [file sensors-24-02643-s001.zip › sensors-2958404-supplementary.pdf]

sensors

MDPI

Electrochemical impedance spectroscopy for the sensing of the kinetic parameters of engineered enzymes

Adriána Dusíková[1], Timea Baranová[2], Ján Krahulec[1], Olívia Dakošová[2], Ján Híveš[2], Monika Naumowicz[3], and Miroslav Gál[2,*]

[1] Dept. of Molecular Biology, Faculty of Natural Sciences, Commenius University, Ilkovičova 6, 842 15 Bratislava, Slovakia
[2] Dept. of Inorganic Technology, Faculty of Chemical and Food Technology STU in Bratislava, Radlinského 9, 812 37 Bratislava, Slovakia
[3] Faculty of Chemistry, University of Białystok, ul. K. Ciołkowskiego 1K, 15-245 Białystok, Poland
[*] Correspondence: miroslav.gal@stuba.sk; (M.G.)

S1: The list of primers for side-specific mutagenesis.

XhoI hEKL F – GGGGCTCGAGAAAAGAGAGGCTGAAGCTATTGTTGGAG

NotI hEKL R - GGGGGCGGCCGCTAATGATGGTGATGGTGGTGCAAGA

Mut hEKL K99R F – CCTCATTATAACAGAAGAAGAAGAGATAATGATATTGCTATG

Mut hEKL K99R R – CATAGCAATATCATTATCTCTTCTTCTTCTGTTATAATGAGG

Mut hEKL N101S F – CATTATAACAGAAGAAGAAAAGATAGTGATATTGCTATGATGCATTTGG

Mut hEKL N101S R – CCAAATGCATCATAGCAATATCACTATCTTTTCTTCTTCTGTTATAATG

Mut hEKL N95E F - GTTATTAACCCTCATTATGAGAGAAGAAGAAAAGATAATG

Mut hEKL N95E R - CATTATCTTTTCTTCTTCTCTCATAATGAGGGTTAATAAC

Mut hEKL N95D F - GTTATTAACCCTCATTATGACAGAAGAAGAAAAGATAATG

Mut hEKL N95D R - CATTATCTTTTCTTCTTCTGTCATAATGAGGGTTAATAAC

Mut hEKL D100N F – CATTATAACAGAAGAAGAAAAAATAATGATATTGCTATGATGC

Mut hEKL D100N R - GCATCATAGCAATATCATTATTTTTTCTTCTTCTGTTATAATG
